# Pharmacologic reprogramming of virus-tumor crosstalk enhances measles virus antitumor activity in BRAF mutant colorectal cancer models

**DOI:** 10.1186/s13046-026-03749-9

**Published:** 2026-06-06

**Authors:** Valery Chavez, Melissa Montero, Ngoc Huong Giang Tran, Carolina Merchan Mendes, Yuguang Ban, Rimpi Khurana, Michelle Ceccarelli, Jaime R. Merchan

**Affiliations:** 1 Department of Medicine, Division of Medical Oncology, University of Miami Miller School of Medicine, Sylvester Comprehensive Cancer Center, Miami, FL USA; 2 University of Miami College of Arts and Sciences, Miami, FL USA; 3 Department of Public Health Sciences, Division of Biostatistics, Sylvester Comprehensive Cancer Center, University of Miami Miller School of Medicine, Miami, FL USA

**Keywords:** Oncolytic virotherapy, Colorectal cancer, BRAF mutation, Measles virus, Minnelide, Molecular mechanisms, Tumor stroma

## Abstract

**Background:**

Oncolytic viruses are promising cancer biotherapies but often show limited durability as single agents due to tumor-intrinsic resistance mechanisms. Because therapeutic efficacy is shaped by dynamic virus–tumor–stromal interactions, pharmacologic strategies that reprogram this interface may enhance virotherapy responses. Here, we investigate whether targeting tumor stress and survival pathways with triptolide and its prodrug Minnelide can enhance the oncolytic efficacy of measles virus in colorectal cancer.

**Methods:**

The in vitro effects of CD46 targeted (MV-GFP) and dual targeted (MV-CD46-muPA) oncolytic MV, alone and with Triptolide were assessed in human CRC cell lines. Mechanistic studies included gene expression analysis, functional proteomics, and western blotting. In vivo efficacy of MV-CD46-muPA combined with Minnelide was evaluated in HT29 and HCT116 xenografts. Biological effects were further characterized using transcriptomic profiling (RNA-seq), targeted gene expression (NanoString), and histological analysis.

**Results:**

Triptolide enhanced MV mediated oncolysis in vitro, particularly in BRAF V600E mutant CRC cell lines, and modulated key cancer pathways including AKT, apoptosis and metabolism. In vivo, Minnelide significantly improved the efficacy of systemically administered MV-CD46-muPA in human CRC xenografts, with greater effects in BRAF mutant (HT29) models. The combination modulated cell cycle, metabolism, and survival associated genes, promoted apoptosis, and improved intratumoral viral distribution. These molecular effects resulted in reduced tumor cell proliferation (Ki67), decreased angiogenesis (CD31), and increased apoptosis (TUNEL) relative to single agents.

**Conclusion:**

Triptolide and Minnelide significantly enhance the oncolytic efficacy of measles virus in colorectal cancer, particularly in BRAF-mutant tumors in vivo, through reprogramming of virus–tumor–stromal interactions. This reprogramming arises from coordinated modulation of survival, metabolic, and stromal signaling pathways and is accompanied by improved intratumoral viral distribution. These findings position virus–tumor–stromal crosstalk as a central mechanistic axis of virotherapy response and provide a strong rationale for the translational and clinical development of rational virus–drug combination strategies in colorectal cancer and other malignancies.

**Supplementary Information:**

The online version contains supplementary material available at https://doi.org/10.1186/s13046-026-03749-9.

## Background

Advanced colorectal cancer (CRC) remains a leading cause of cancer mortality worldwide, with approximately 53,000 deaths projected in the United States in 2025 [1]. Therapeutic resistance remains a major barrier to cures, driven by the aggressive biology of microsatellite-stable (MSS) disease and frequent KRAS mutations. This challenge is especially pronounced in tumors with BRAF V600E mutations, which are associated with poor prognosis despite targeted therapies [2, 3]. Additionally, the dense stromal microenvironment of advanced CRC limits drug penetration and further reduces the efficacy of systemic treatments [4, 5]. These factors highlight an urgent need for novel biotherapies capable of overcoming both tumor intrinsic and stromal resistance mechanisms.

Oncolytic viruses (OVs) offer a promising platform for cancer therapeutics due to their dual ability to selectively lyse tumor cells and stimulate antitumor immunity [6, 7]. Among OVs, measles virus (MV) has shown preclinical and clinical safety and antitumor activity in hematological malignancies and solid tumors [8–12], alone and in combination with targeted and immunotherapy agents [13–15]. To improve specificity and overcome stromal barriers, we previously engineered urokinase receptor (uPAR) retargeted oncolytic MV vectors [16], leveraging high uPAR expression in CRC and its stroma [17] to enhance viral entry. These vectors have demonstrated safety and efficacy in CRC and breast cancer models [6, 16, 18–20]. More recently, our dual targeted MV-CD46-muPA, which engages human tumor cells via CD46, and mouse stroma via murine uPAR, has achieved superior antitumor activity compared with single-targeted viruses [21].

Despite these advances, the clinical impact of OVs as monotherapies remains limited [22], largely due to tumor evasion mechanisms such as restricted viral entry, antiviral immune responses, interferon signaling activation, epigenetic inhibition of viral transcription, and APOBEC mediated resistance [23–27]. This highlights the need for novel virus-drug combinations to overcome resistance and improve oncolytic efficacy in vivo.

Triptolide (TRP), a diterpenoid triepoxide derived from Tripterygium Wilfordii (thunder god vine) exhibits potent antitumor activity through modulation of multiple oncogenic pathways, inducing ER stress, apoptosis, and antiangiogenic effects [28]. Its water-soluble form Minnelide (MN) is currently in phase 1 and 2 clinical trials in solid and hematological malignancies and has demonstrated synergy with targeted and chemotherapeutic agents. TRP and MN induce cytotoxic effects against CRC in vitro [29, 30], and enhance tumor delivery of anticancer agents [31–34], which makes it a promising agent for virus-drug combinations. Here, we investigate the therapeutic potential and molecular mechanisms of MV vectors in combination with TRP and Minnelide in preclinical models of colorectal cancer.

## Materials and methods

### Cell culture and reagents 

HT29 (ATCC #HTB-38), HCT116 (ATCC #CCL-247), HCT15 (ATCC #CCL-225), RKO (ATCC #CRL-225), NCI-H508 (ATCC # CCL-253), and LoVo (ATCC #CCL-229) cells were purchased from the American Type Culture Collection (ATCC, Manassas, VA). Cells were tested for mycoplasma prior to the experiments. Cells were maintained in Corning™ McCoy’s 5 A (HT29 and HCT-116), RPMI (HCT-15 and NCI-H508), F-12 K (LoVo) and EMEM (RKO) containing 10% fetal bovine serum (FBS), penicillin, and streptomycin at 37 °C and 5% CO_2_. Cells were used from passages 1 to 6. Triptolide (TRP) was obtained from Sigma Chemical (St. Louis, MO, USA). Minnelide (MN) was obtained from Minneamrita Therapeutics (Tampa, FL, USA). Oncolytic measles virus (MV) vectors MV-GFP (targeting CD46) and MV-CD46-muPA (dual targeted virus against CD46 and murine uPA receptor) were generated and propagated as [21, 35].

### In vitro viral replication 

Cancer cells (2 × 10^5^ per well) were infected with MV-GFP or MV-CD46-muPA MOI of 3 for two hours (37 °C, 5% CO_2_). Unbound virus was removed, and cells were cultured in DMEM/10% FBS with or without triptolide (6.25nM). RNA was collected at specified timepoints using the RNeasy Plus Mini Kit (Qiagen). RNA quality and concentration were assessed using a NanoDrop ND-1000 spectrophotometer (ThermoFisher Scientific). cDNA was synthesized from 1 µg RNA using the High-Capacity cDNA Reverse Transcription Kit (Applied Biosystems, USA). qPCR was performed on a CFX96 Touch Real-Time PCR Detection System (Bio-Rad) using RT^2^ SYBR Green qPCR Mastermix (Qiagen) and specific primers: *MV-N* gene forward: GAGAAGCCAGGGAGAGCTCACG, reverse: GGGCAGCTCTCGCATCAC; *GAPDH* gene forward: ACATCGCTCAGACACCATG, reverse: TGTAGTTGAGGTCAA TGAAGGG. cDNA was amplified using 20ng of cDNA in a final reaction volume of 20uL. Cycling conditions were: 1 cycle of initial denaturation at 95 °C for 10 min followed by 40 cycles of denaturation at 95 °C for 15s and annealing at 60 °C for 1 min. Gene expression was normalized to GAPDH using the ΔΔCT method, and values were normalized to MV-only condition, which was set to 1. Results were expressed as relative change to virus-only treatment [36].

### In vitro cell cytotoxicity

Cancer cells were seeded at a density of 1.2 × 10^5^ cells/well in 6-well plates, kept in culture overnight and then infected with MV vectors at MOI= 1 for two hours, (37 °C, 5% CO_2_), after which plates were washed with PBS to remove unbound virus. Infected cells were then trypsinized and seeded with or without TRP in xCelligence 96 well plates. Real time cell index was measured using the xCelligence Real Time Cell Analysis (RTCA) MP (ACEA Biosciences). For single agent TRP and MV experiments, cells were plated as above, and treatment was added at escalating doses before cell seeding into xCelligence plates. Experiments were performed at least in quadruplicates and repeated twice.

### 3D tumor spheroid assay

Three-dimensional tumor spheroids were generated using previously described protocols [37, 38], with modifications. Human colon cancer cells were seeded at a density of 3,000 cells per well in round-bottom, cell-repellent 96-well plates (CELLSTAR^®^, Greiner Bio-One, Cat# 650970). Plates were centrifuged using a 5804R centrifuge (Eppendorf) at 1,200 rpm for 3 min at 4 °C to facilitate cell aggregation and then cultured for 72 h to allow spheroid formation. Spheroids were maintained in complete culture medium –appropriate for each colon cancer cell line (as above- supplemented with 2.5% growth factor-reduced Matrigel (Corning, CA Cat# 354230). For treatment studies, spheroids were infected with virus at a multiplicity of infection (MOI) of 5 and incubated at 37 °C for 16 h. The viral dose was selected based on preliminary optimization experiments demonstrating reproducible 3D spheroid infection at this MOI. Following viral infection, Triptolide was added at a final concentration of 6.25 nM. Six days post-treatment, cell viability was determined using the homogeneous CellTiter-Glo^®^ 3D Cell Viability Assay (Promega, Cat. No. G9682). The assay reagent was added directly to each well according to the manufacturer’s protocol to achieve complete spheroid lysis, and total luminescence was measured.

### Reverse Phase Protein Array (RPPA) analysis

Functional proteomic analysis of treated and untreated HT29 cells was performed at the RPPA Core Facility at MD Anderson Cancer Center (Houston, TX). Treatments consisted of vehicle, virus (MV-GFP MOI= 0.5), triptolide (6.25nM) and combination, for 48 h. Antibodies and approaches are available online (https://www.mdanderson.org/research/research-resources/core-facilities/functional-prote omics-rppa-core.html) [39]. RPPA data generation and normalization (in log2 scale) were performed by the Core. Differential protein expression was analyzed using the “limma” R package (v3.26.9), with significance defined as *p* < 0.05 and FDR < 0.2. Enrichment analyses were performed by the Sylvester Cancer Center Bioinformatics Core facility using Ingenuity Pathway Analysis (IPA^®^, QIAGEN).

### Western blot analysis

HT29 cells (1.2 × 10^6^) were plated in 10 cm Petri Dishes and treated with MV-GFP MOI= 0.5 with or without TRP 6.25nM. After 48 h of treatment, cells were collected and lysed for western blot analysis. Protein concentrations were determined using Pierce™ Bradford Plus Protein Assay Reagent (Thermo Scientific, Rockford IL). 20–30 µg of protein lysates were loaded into 4–20% Mini-Protean TGX gels (BioRad), transferred to 0.45 μm pore sized Nitrocellulose membranes (ADVANTEC) and probed with the following primary antibodies: LC3A/B #3868 (1:200), PARP #9542 (1:100), ASNS (1:200) #92,479, p53 #48,818 (1:800), RRM2 #65,939 (1:500), Cleaved Caspase 8 # 9496 (1:800), PERK #5386 (1:200), GYS # 3896 (1:200) from Cell Signaling Technologies MA, USA. Also tested were Phospho-Akt Ser473 #81,283 (1:300), Phospho-S6 #2571 (1:200) and GAPDH #181,602 (1:10000) from Abcam Limited, Cambridge, UK. Experiments were performed at least in duplicated. Band intensities were quantified by densitometric analysis using ImageJ software (NIH, Bethesda, MD) after background subtraction and normalized to GAPDH as a loading control.

### Animal studies

Animal studies were approved by the University of Miami Institutional Animal Care and Use Committee. Five- to six-week-old female and male NSG mice (Jackson labs, Farmington, CT) were implanted with HT29 (1.5 × 10^6^) or HCT116 (3 × 10^6^) cells subcutaneously into the right flank. Treatment started when tumors reached the target volume (> 40–60 mm^3^, for early tumors, and *≥* 150 mm^3^, for advanced tumors). Depending on the experiment, mice (*n* = 8–10/group) were treated with Minnelide (0.1, 0.21 or 0.42 mg/kg, via intraperitoneal administration) for 21 days, MV-CD46-muPA (1.5 × 10^6^ TCID_50_), via tail vein (every other day x 3), or the combination of MV-CD46-muPA (as above) and Minnelide for 21 days.

Tumor volume was measured twice a week and calculated with the following formula (Width^2^ × Length × 0.5). Clinical signs of toxicity were continuously monitored. Animals were followed until they reached sacrifice criteria (when tumor burden reached 10% of body weight, tumor ulceration occurred, or mice became moribund).

### Tumor correlative studies

Five- to six-week-old female and male NSG mice (Jackson labs, Farmington, CT) were implanted with 1.5 × 10^6^ HT29 cells subcutaneously into the right flank. When tumors reached a volume of *≥* 150 mm^3^, mice were randomized and treated with vehicle, MV-CD46-muPA (1.5 × 10^6^ TCID_50_), via tail vein 2 times every other day alone and in combination with Minnelide 0.21 mg/kg for 7 days. After the last Minnelide treatment (day 7), tumors were dissected and divided into two halves, for RNA extraction and tumor immunofluorescence studies, respectively.

### RNA sequencing and targeted gene expression analysis

RNA was isolated from (treated and control) HT29 tumor homogenates using the KingFisher Apex system (ThermoFisher) and Zymo Direct-zol Mag-Bead chemistry, and RNA integrity was confirmed on an Agilent Fragment Analyzer. High-quality RNA was submitted to Novogene, where poly-A–selected, non-directional libraries were constructed and sequenced on a NovaSeq X Plus platform. Sequencing reads were processed using Xenome for xenograft host–tumor separation, aligned with STAR, and normalized using variance-stabilizing transformation. Differential expression analysis was performed with DESeq2 (adjusted *p* < 0.05, |log2FC|>1), followed by GO, KEGG, and Ingenuity Pathway Analysis to identify enriched biological pathways (see supplementary methods).

Targeted gene expression profiling was performed using the NanoString nCounter XT Human Tumor Signaling 360 panel on RNA extracted from HT29 cells and tumors. Data were processed using nSolver 4.0 and the Advanced Analysis module with standard normalization and Benjamini–Hochberg correction applied. Heatmaps were generated from significantly regulated genes and pathway-defined gene sets. Full experimental details for RNA-seq and NanoString workflows are provided in the Supplementary Methods.

### Tumor immunofluorescence studies

Tumor samples were collected and frozen in OCT. After sectioning, slides were fixed with precooled (-20 °C) acetone for 10 min, washed and blocked with 10% goat serum. Sections were incubated with antibodies against MV-nucleoprotein (2 µg/ml; Sigma Aldrich), Ki67 (1:500; Abcam Limited, Cambridge, UK Ab15580), CD31(1:25, Abcam Limited, Cambridge, UK Ab182981) and FAP (1:500, Invitrogen by Thermofisher Scientific). Slides were then washed (PBS) and incubated with Alexa Fluor 488 or Alexa Fluor 555 (for anti-MV-nucleoprotein) 1:350 for 30 min, washed and mounted with antifade mounting medium and imaged by fluorescent microscopy.

In vivo apoptosis was assessed by the Terminal deoxynucleotidyl transferase dUTP nick end labeling *(TUNEL)* assay, as previously reported [21] Briefly, tumor slides were washed twice with PBS (after fixation), permeabilized with 0.2% TritonX-100 for 20 min at room temperature and after 2 additional washes with PBS, sections were probed with label solution (for negative controls) or TUNEL reaction mix, following manufacturer’s instructions (In Situ Cell Death Detection Kit-TMR red; Roche Applied Science, Indianapolis, IN). Fluorescent images were captured with the Leica Stellaris 5 using LAS X software. All treatment groups were imaged with the same settings and underwent the same image processing. Images were analyzed using NIS Elements AR (Nikon) software. Mean fluorescence intensity (MFI) for each image was quantified using the binary-threshold tool in NIS-Elements AR (Nikon) software. MFI was assessed in 4–5 randomly selected, non-overlapping image fields from viable tumor regions per tumor section. For Ki67 and TUNEL analysis, percent of positive fluorescent staining was analyzed as described in supplementary methods. At least three tumor sections were analyzed per treatment group. All images were acquired using identical exposure settings across treatment groups.

To quantitatively assess TUNEL and Ki67 staining at the single-cell level, cells were segmented from the DAPI channel using Fiji (ImageJ) by applying local maxima–based detection followed by particle analysis to identify individual nuclei. Prior to segmentation, images were preprocessed with background subtraction and smoothing to improve nuclear boundary definition. For each detected nucleus, the mean fluorescence intensity of the TUNEL (red) channel was quantified after background correction and flat-field normalization. A data-driven intensity threshold was then determined using Gaussian mixture modeling to classify nuclei as TUNEL positive or negative. The proportion of TUNEL positive nuclei was then calculated.

### Statistical analysis

In vitro data are presented as means +/- SD. Results from in vivo studies are shown as means +/- SEM. All in vitro experiments were performed at least in triplicate unless otherwise specified and repeated at least twice. Statistical analysis among groups was performed by ANOVA, and sub-group comparisons were made with the Tukey-Kramer test, as appropriate. For in vivo tumor growth studies, longitudinal tumor volume measurements were analyzed using linear mixed-effects models to account for repeated measures within individual animals over time, with post hoc pairwise comparisons among treatment groups performed using Tukey–Kramer adjustment for multiple comparisons Overall survival was analyzed by the Kaplan–Meier method and differences were analyzed by the log-rank test. Statistical significance was set at *p* < 0.05.

## Results

### Triptolide potentiates in vitro measles virus oncolysis in colorectal cancer

The effects of TRP on MV-GFP (targeting CD46) and MV-CD46-muPA oncolysis were assessed in BRAF (HT29, RKO) and KRAS mutant (HCT116, HCT15) human colon cancer cell lines. As single agents, TRP exhibited dose dependent cytotoxicity at low nanomolar concentrations (Figure S.1), while both MV vectors produced cell line dependent effects, including growth inhibition, syncytia formation, and oncolytic activity (Figure S2). Transient increases in impedance (cell index) observed in some conditions are consistent with syncytia formation and enhanced cell-electrode contact prior to syncytial disruption and cell death (decrease in cell index).

**Fig. 1:**
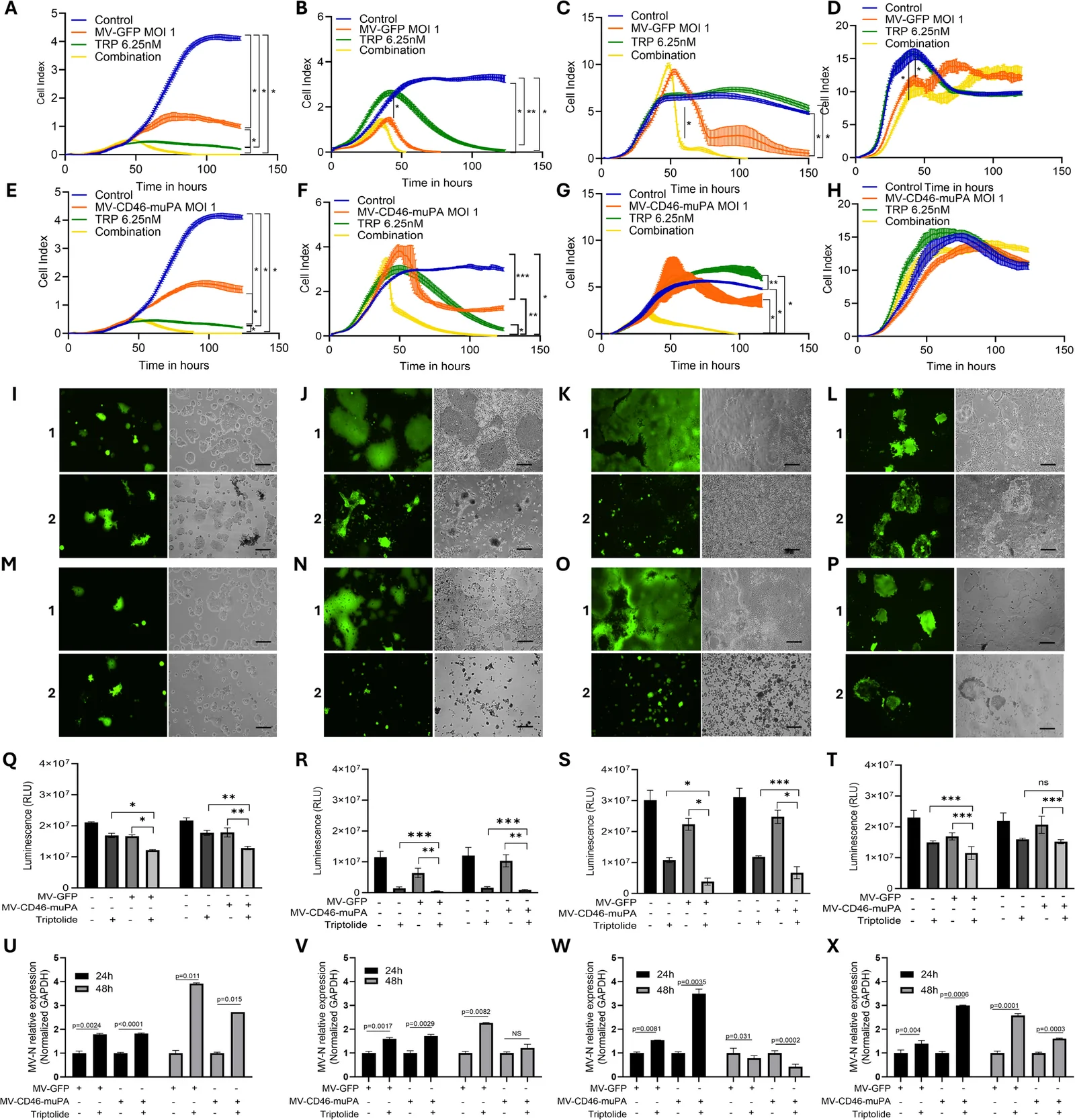
In vitro effects of Triptolide (TRP) on Measles virus (MV) cytotoxicity in human colorectal cancer. HT29 (**A**,** E**), RKO (**B**,** F**), HCT116 (**C**,** G**), and HCT15 (**D**,** H**) cells were treated with Triptolide, MV-GFP (MOI = 1), MV-CD46-muPA (MOI = 1), and their combination, and cell growth was monitored in real time using the xCelligence assay. Cell growth curves represent the average cell index (+/- SD) of experiments performed at least in quadruplicate. *p < 0.001; **p = 0.01, ***p = 0.03. GFP Fluorescence and brightfield images show the effects of MV alone (1) and in combination with TRP (2) in the same cell lines: HT29 **(I**,** M)**, RKO (**J**,** N**), HCT116 **(K**,** O)**, and HCT15 **(L**,** P).** Pictures were taken 48 h post infection. Scale bar: 100 µm). Effects of MV and Triptolide combination on 3D colorectal cancer spheroids. HT 29 (Q), RKO (R), HCT 116 (S), and HCT15 (T) spheroids were established as described in methods and treated with MV-GFP or MV-CD46-muPA (MOI = 5), Triptolide (6.25 nM) or their combinations. Six days after treatment, viability was assessed using the CellTiter-Glo® 3D Cell Viability Assay. Bar graphs represent average luminescence values (RLU ± SD) from triplicate experiments. *p < 0.001, **p < 0.01, ***p < 0.05. Effects of TRP on MV replication in CRC cells. Bar graphs depict differences in viral replication (measured by qRT-PCR) in CRC cell lines at 24 and 48 h after viral infection, comparing virus alone (MOI = 3) and in combination with TRP (6.25 nM). HT29 (**U**), RKO (**V**), HCT116 (**W**), and HCT15 (**X**). Bars represent average (+/- standard deviation) of MV-N expression, normalized for GAPDH. Experiments were done in triplicate

When combined with low concentrations of TRP (6.25 nM), MV-GFP and MV-CD46-muPA significantly enhanced cytotoxicity in HT29, RKO, and HCT116 cells (Fig. 1A-E, B-F, and C-G, respectively). In contrast, HCT15 cells demonstrated relative resistance to both agents, alone or in combination, in this 2D assay (Fig. 1D-H). Microscopy confirmed disruption of MV-induced syncytia in the combination groups for HT29, RKO, and HCT116 cells (Fig. 1. I-M, J-N, and K-O, respectively), whereas HCT15 cells showed less prominent syncytial disruption (Fig. 1. L-P). Combinatorial effects were also observed in additional CRC models, including NCI-H508 cells harboring a non-V600 BRAF mutation (G596R) and KRAS-mutant LoVo cells (G13D) (Figure S3) [40]. In these additional models, MV/TRP effects became more evident at later time points (> 72–96 h), with greater enhancement observed in NCI-H508 cells (Figure S3.A, B) compared with LoVo cells (Figure S3. D, E), especially in the MV-CD46-muPA/TRP combination. Notably, the delayed response kinetics observed in NCI-H508 cells differed from those seen in the HT29 and RKO models, which harbor canonical BRAF V600E mutations, consistent with biological heterogeneity across CRC models. Limited effects were observed with the individual agents alone.

We next evaluated the effects of the virus-drug combination in 3D spheroid models of colorectal cancer. Compared with each agent alone, MV/TRP combinations induced significantly greater cytotoxicity in HT29 (Fig. 1Q), RKO (Fig. 1R), and HCT116 spheroids (Fig. 1S). Comparable findings were also observed in NCI-H508 and LoVo spheroids (Figure S3, C, F). Overall, combinatorial effects in the 3D spheroid models were more consistently observed than in the corresponding 2D assays, including greater sensitivity of HCT15 spheroids to the combination treatments. However, the MV-CD46-muPA/TRP combination did not produce statistically significant differences in cytotoxicity compared with TRP alone (Fig. 1T).

Finally, we assessed the effects of TRP on viral replication. Addition of TRP increased viral RNA at 24 and 48 h in HT29, RKO, HCT116, and HCT15 cells (Fig. 1. U, V, W and X). At 72 h, extensive cytotoxicity (cell and syncytia disruption) precluded reliable MV-RNA quantification (data not shown). This observation was evident in HCT116 cells, where TRP increased MV-N RNA at 24 h but not at later time points, due to marked cytotoxic effects (Fig. 1.O).

### Molecular mechanisms of TRP mediated amplification of viral oncolysis

To elucidate the molecular basis of the observed treatment interaction, we performed targeted gene expression (Nanostring) and functional proteomic (RPPA) analysis in BRAF mutant HT29 cells treated with MV, TRP or the combination for 48 h. Because MV-GFP and MV-CD46-mUPA target human colon cancer cells similarly via CD46, and TRP enhanced oncolysis in both viral vectors, MV-GFP was used for mechanistic studies. A viral MOI of 0.5 was used to minimize extensive virus-induced cytopathic effects and syncytia formation -especially with the combination therapy- while preserving RNA and protein integrity for molecular analyses. An MOI = 0.5 were used for the mechanistic studies.

### Gene expression profiling

**Fig. 2:**
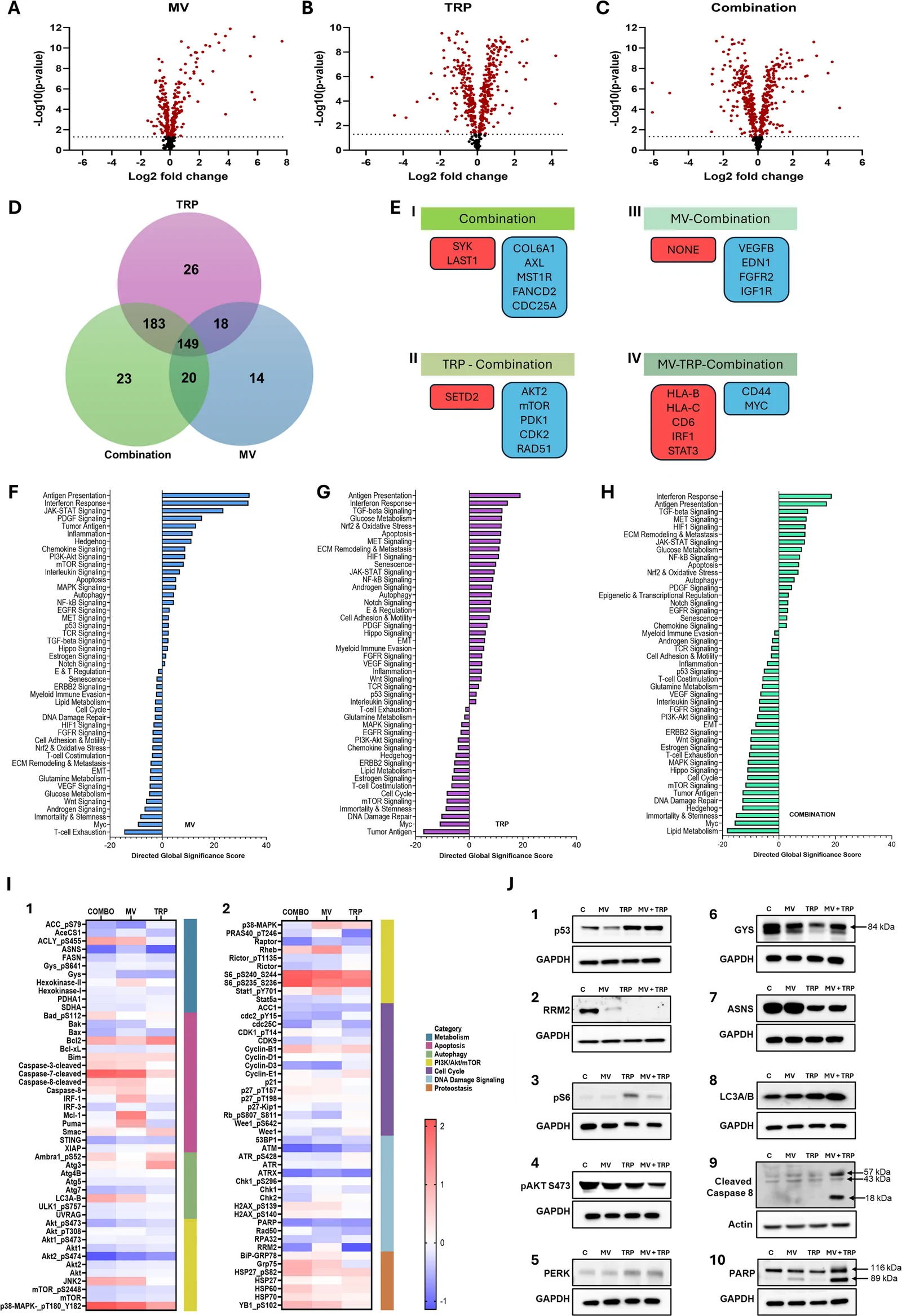
In vitro molecular effects of MV, Triptolide (TRP) and the virus-drug combination in BRAF V600E mutant colon cancer (HT29).** A-C** Volcano plots showing differentially expressed genes (DEGs) from HT29 cells treated with MV (MOI = 0.5), TRP (6.25 nM) and Combination compared to control group. **D** Venn diagram showing common and unique genes across the treatment groups. **E** Representative upregulated (red) and downregulated (blue) genes unique to the virus-drug combination group (**I).** Commonly up- and downregulated genes in the TRP and combination (**II**), MV and Combination (**III**), and all three (MV, TRP, and Combination) treatments groups (**IV**). **F-H** Pathway enrichment analysis of DEGs in each treatment group compared to control. The X-axis indicates the Direct global significance score, and the Y-axis shows the list of the top enriched pathways.** I**. Functional proteomic analysis (RPPA) of HT29 cell treated with single agents and combination. **1, 2** Heatmap of selected statistically significant differentially expressed proteins in each treatment group, clustered by pathway category. A color gradient scale bar was used to indicate the magnitude and direction of the protein changes (red shades represent upregulation-positive Log2FC, blue shades represent downregulation-negative Log2FC values and white indicated no significant changes). **J** Protein lysates were obtained from control and treated HT29 cells after 48 h for western blot analysis (**1**: p53, **2**: RRM2, **3**: pS6, **4**: pAkT S473, **5**: PERK, **6**: GYS, **7**: ASNS, **8**: LC3A/B, **9**: Cleaved Caspase 8; and **10**: PARP), GAPDH or Actin were used as loading controls

Volcano plots (Fig. 2. A-C) and Venn diagram (Fig. 2. D and Supplementary Data) illustrate the spectrum of and overlap of differentially expressed genes by single agents and the combination, as well as representative common and unique genes in the combination treatment (Fig. 2. E). All three treatments commonly modulated 149 genes. Notably, the combination shared more transcriptional changes with TRP (183 genes) than with MV alone (20 genes), suggesting TRP drives the transcriptional landscape. The combination uniquely regulated 23 genes, compared to 26 genes in the TRP group and 14 genes in the MV treated group. Pathway analysis showed that MV (Fig. 2. F) and TRP (Fig. 2. G) independently down regulated cell cycle, DNA damage repair, Myc, lipid and glutamine metabolism pathways, while upregulating apoptosis, autophagy, NF-kB and JAK/STAT signaling in vitro. The virus-drug combination induced broader transcriptional suppression, particularly of PIK3/mTOR, MAPK, WNT, hedgehog, stemness, EMT, lipid and glutamine metabolism, and angiogenic (VEGF, FGF) pathways (Fig. 2. H). Although interferon stimulated genes were induced in all conditions, the degree of upregulation was attenuated in the combination compared to MV alone.

### Functional proteomics

RPPA analysis revealed that MV alone activated antiviral/stress responses (STAT1_p_Y701, H2AX_p_S139), enhanced translational activity (ribosomal S6_pS240_S2244/pS235-S236), and induced caspase (cleaved CASP3/7/8) activation and PUMA upregulation (Fig. 2. I, 1–2 and Supplementary Data). On the other hand, several survival programs were maintained, with no statistically significant changes in proteins involved in DNA repair (CHK1/CHK1_p_S345, CHK2/CHK2_p_T68, RRM2, ATR/ATR_p_S428) and proteostasis (HSP70, HSP27, GRP78, and YB1). AKT phosphorylation was reduced, without a significant decrease in mTOR. Triptolide downregulated AKT2_p_S474 activity, increased BIM and SMAC, and inhibited hexokinase 1, fatty acid synthase (FASN) and asparagine synthetase (ASNS), suggesting metabolic remodeling. Effects on chaperone, caspases, and PUMA were limited.

The MV-TRP combination integrated and amplified these effects. It abrogated viral compensatory mechanisms (IRF1, IRF3 and STAT5A), while amplifying cell death (Fig. 2. I. 1–2). AKT, mTOR and RAPTOR activity was suppressed. DNA repair capacity collapsed (ATM, CHEK1, CHEK2, PARP, RPA32, RRM2, and ATRX). Autophagy was disrupted (Decreased ATG7/5/4B, ULK1_p_S757 despite, LC3 accumulation). Importantly, apoptosis was reinforced (XIAP downregulation; BIM, SMAC upregulation, and caspase activation Fig. 2. I, 1).

TRP and MV reprogrammed tumor metabolism, as demonstrated by significant down regulation of hexokinase-1, glycogen synthase, pyruvate dehydrogenase E1 alpha (PDHA1), and succinate dehydrogenase complex subunit A (SDHA). Similar to TRP, the combination also downregulated FASN and ASNS. Western blot analysis for selected proteins confirmed the above findings. As shown in Fig. 2. J, key proteins involved in cancer signaling pathways were differentially regulated in the combination group: RRM2, pS6, p-AKT, ASNS, GYS were suppressed, while LC3A/B, p53, PERK, Cleaved Caspase 8 and cleaved PARP levels were increased in the TRP-MV combination.

### In vivo antitumor effects of Minnelide and MV-CD46-muPA in early and advanced colorectal cancer xenografts

To extend these findings in vivo, MV-CD46-muPA and Minnelide were evaluated in HT29 (BRAF mutant) and HCT116 (KRAS mutant) xenografts. The dual targeted MV-CD46-muPA measles viral vector was selected to exploit its ability to infect both human tumor cells via CD46 and murine stromal cells via uPAR [21].

First, HT29 tumor bearing mice were treated with escalating (0.21 mg/kg and 0.42 mg/kg) doses of single agent Minnelide, once tumors reached approximately 50mm^3^. As shown in Figure S.4, Minnelide significantly delayed tumor growth and improved overall survival in a dose dependent manner. To enhance translational applicability and balance safety and efficacy for combination studies, the 0.21 mg/kg dose was selected for subsequent experiments in this model.

**Fig. 3:**
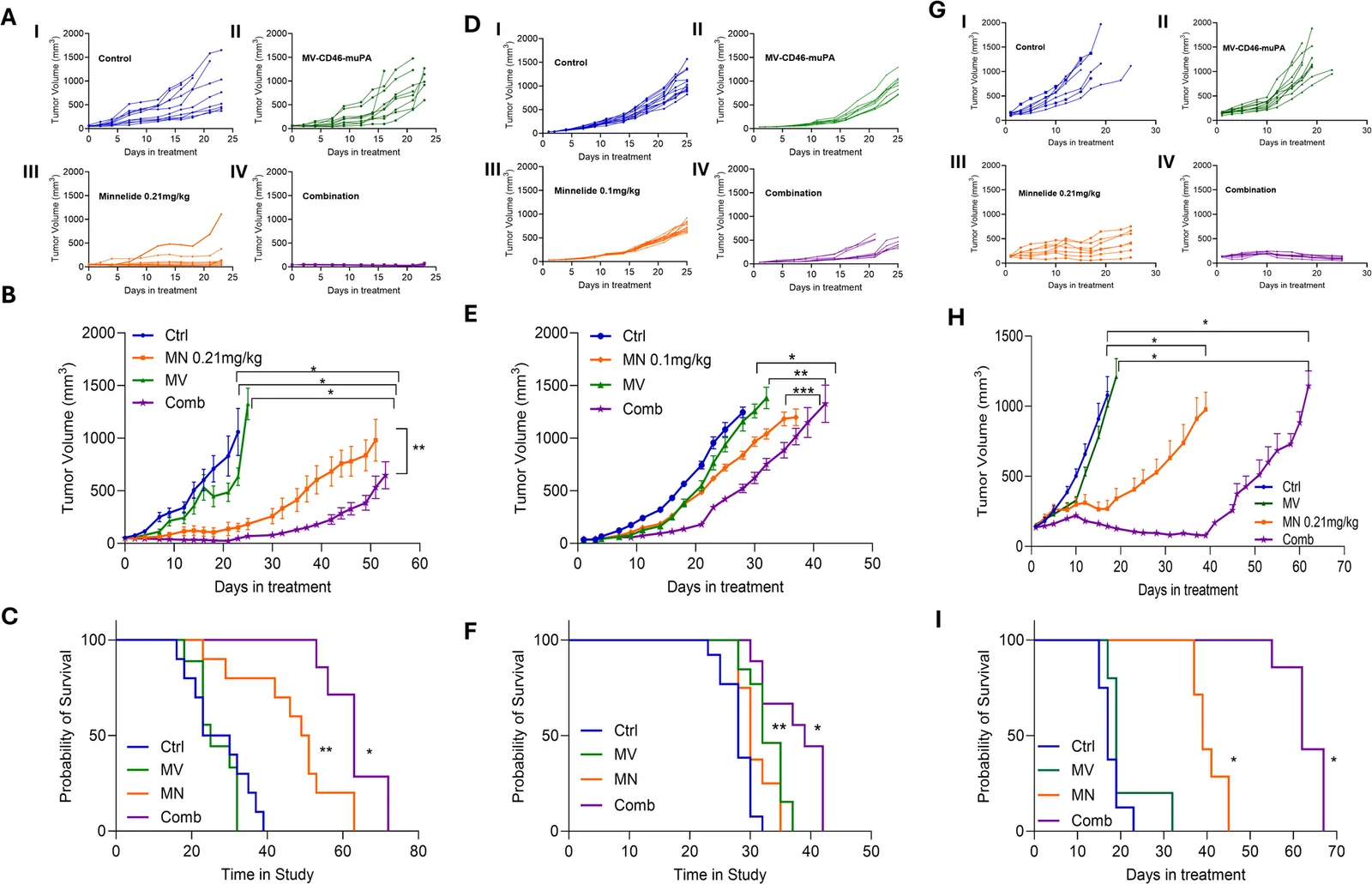
In vivo antitumor effects of Minnelide in combination with measles virus in BRAF and KRAS mutant colorectal cancer xenografts. Top row: Spider plots showing individual tumor growth trajectories in early-stage HT29 xenografts (initial tumor volume >40–60 mm³; **A**, HCT116 xenografts (**D**), and advanced HT29 xenografts (initial tumor volume >150 mm³; **G** treated with Control (**I **), MV-CD46-muPA (**II**), Minnelide (**III**), or the combination (**IV**). Middle row: Average tumor growth curves for early-stage HT29 (**B**), HCT116 (**E**), and advanced HT29 xenografts (**H**) treated with vehicle, single agents, or combination therapy. Bottom row: Kaplan–Meier survival analyses for treated and control animals. **C** Early-stage HT29 xenografts: median survival was 26.5 days (95% CI: 16–35) for control, 25 days (95% CI: 18–32) for MV-CD46-muPA, 50 days (95% CI: 23–53) for Minnelide, and 63 days (95% CI: 53–72) for combination therapy. **F** HCT116 xenografts: median survival was 28 days (95% CI: 25–30) for control, 30 days (95% CI: 28–35) for MV-CD46-muPA, 32 days (95% CI: 30–35) for Minnelide, and 32 days (95% CI: 30–32) for combination therapy. **I** Advanced HT29 xenografts: median survival was 17 days (95% CI: 15–19) for control, 19 days (95% CI: 17–19) for MV-CD46-muPA, 39 days (95% CI: 37–45) for Minnelide, and 62 days (95% CI: 55–67) for combination therapy. Statistical analysis: Early-stage HT29: **B** *p<0.0001, **p=0.034; **C** *p<0.0001, **p=0.0002. HCT116: **E** *p<0.0001, **p=0.0035, ***p=0.0014; **F** *p<0.0001, **p=0.0009. Advanced HT29: **H** *p<0.0001; **I** *p<0.0001

Next, HT29 tumor bearing mice were randomized to receive vehicle, MV-CD46-muPA alone (3 doses, intravenously), Minnelide (intraperitoneally, for 21 days), or the combination. While single agents delayed tumor progression, the virus-drug combination produced a more pronounced and durable antitumor response and significantly extended survival (Fig. 3.A-C). No treatment related deaths or signs of clinical toxicity were observed.

To determine whether the effects of the MV-MN combination is influenced by the oncogenic context, HCT116 xenografts (KRAS mutant) were treated using the same dosing scheme. In this model, Minnelide (0.21 mg/kg) produced robust tumor growth inhibition that was comparable to the combination (Figure S5). To explore dose dependent combinatorial effects, additional studies tested lower Minnelide doses (0.1 mg/kg) alone and in combination with MV-CD46-muPA. At this lower dose, the combination treatment was associated with superior growth delay and survival compared to single agents and controls (Fig. 3. D-F), although the effect size was more modest than in the BRAF mutant model in this setting.

Finally, we assessed the effects of the MV-Minnelide combination in a more advanced disease context, using HT29 xenografts with larger tumor burden (> 150mm^3^). Treatment dosing and schedules were similar to experiments in smaller HT29 tumors. As shown in Fig. 3. G-I, the combination achieved superior efficacy compared to single agents, with clear reductions in tumor size, significant survival improvement, and no visible toxicity.

### In vivo molecular and biological effects of MV-CD46-m-uPA and Minnelide in BRAF mutant colorectal cancer

To define the molecular and histological correlates of therapeutic response, we performed comprehensive RNA sequencing and targeted gene expression analyses on tumors harvested from treated HT29 xenografts.

### Global transcriptomic landscape

**Fig. 4:**
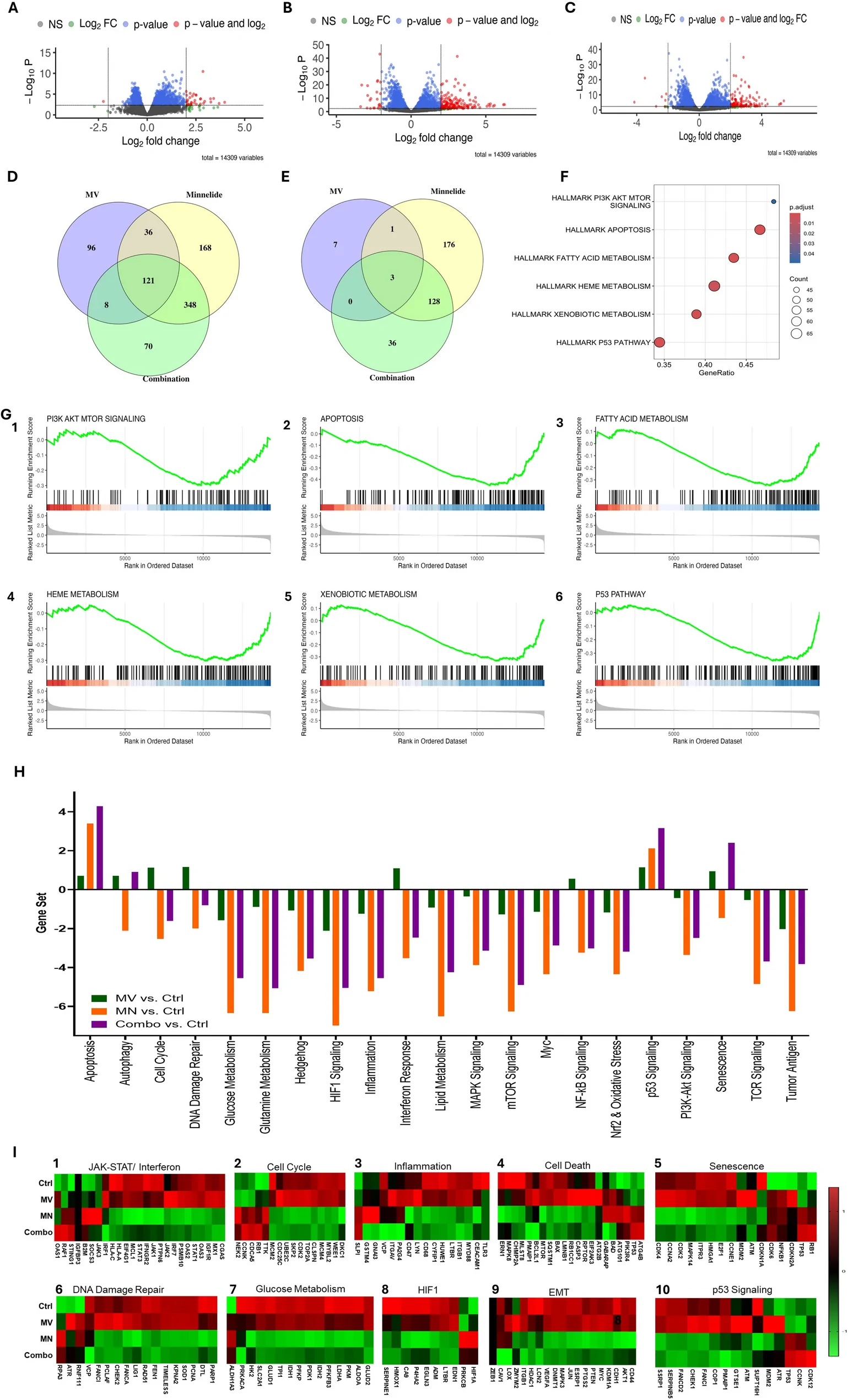
Effects of MV and Minnelide on HT29 in vivo gene expression. **A**-**C**. RNA seq: Volcano plots showing differentially expressed genes for MV, Minnelide, and Combination groups vs. control, respectively (x-axis: log2 fold change; y-axis: –log10 p-value; dashed lines: ±2-fold change, *p* = 0.005). Red, green, and blue points meet both, fold change only, or p-value only thresholds, respectively. **D-E.** Venn diagrams showing up (**D**)- and down- (**E**) regulated common and unique genes in each treatment group. **F.** GSEA of combination vs. control ranked genes, showing NES for hallmark gene sets. **G.** Representative enrichment plots: green line = running score; red/blue bars = leading-edge genes; black lines = all gene set members. **1** PI3K-AKT-mTOR signaling, **2** Apoptosis, **3** Fatty acid metabolism, **4** Heme metabolism, **5** Xenobiotic metabolism, **6** P53 pathway). **H**. Nanostring: Pathway enrichment analysis of DEGs in each treatment group vs. control. The X-axis indicates the list of the top enriched pathways, and the Y-axis shows the enrichment significance score. **I.** Heatmaps of significant differentially expressed pathways. **1** JAK-STAT/Interferon, **2** Cell cycle, **3 **Inflammation, **4 **Cell death, **5** Senescence, **6** DNA Damage repair, **7** Glucose metabolism,** 8** Hypoxia inducible factor (HIF-1), **9** Epithelial to mesenchymal transition (EMT), **10** p53 signaling.

Principal component analysis (PCA) revealed distinct separation between control and treatment groups, with Minnelide and the combination clustering closely together, consistent with overlapping transcriptional profiles (Supplementary Fig. S.6.A). The magnitude and significance of gene expression changes across treatments are illustrated in the volcano plots, shown in Figs. 4. A–C. Differential expression analysis identified 272 genes altered in the MV group, 981 in Minnelide, and 714 in the combination (adjusted *p* < 0.05, |log₂FC| ≥ 1). A core set of 124 genes was shared among all three treatments, while 106 were specific to the combination (Fig. 4D-E and Supplementary Data). Pathway enrichment (GSEA) showed that the combination treatment significantly suppressed six hallmark pathways Apoptosis, P53 Pathway, Fatty Acid Metabolism, Xenobiotic Metabolism, Heme Metabolism, and PI3K/AKT/mTOR Signaling (Fig. 4. F) indicating reduced metabolic activity and increased stress/damage response. All six pathways exhibited negative normalized enrichment scores (NES < 0), indicating suppression relative to control, consistent with reduced metabolic activity and enhanced stress- and damage-response signaling. Representative enrichment plots for these pathways are shown in Fig. 4. G 1–6, depicting the running enrichment score (green line) and the distribution of leading-edge genes contributing to pathway modulation.

Ingenuity Pathway Analysis (IPA) was conducted using all significantly differentially expressed genes (log2FC > 1, adjusted p-value < 0.05) that overlapped across all treatments (124 genes), as well as those specific to each treatment: MV (103 genes), Minnelide (344 genes), and Combination (106 genes). The resulting enriched disease and biological function categories are shown in Supplementary Figure S.6B (MV), S.6 C (Minnelide), and S.6D (Combination). Across the three treatment-specific gene sets, six categories were shared by all groups (Cancer, Cell Cycle, Cell Death and Survival, Inflammatory Disease, Inflammatory Response, and DNA Replication, Recombination, and Repair), while additional categories were enriched in only one or two groups. The Minnelide-specific set produced the highest Fisher’s exact enrichment scores (–log[p-value]), Figure S6C), reflecting the broad transcriptional impact of Minnelide and the increased statistical power conferred by its larger gene set (344 genes). The Combination-specific set, although smaller (106 genes) and therefore associated with a lower statistically significant –log(p-value) (Figure S6D), engaged the same core categories and additionally enriched Cell-mediated Immune Response and Antigen Presentation (Fig. S6D), reflecting MV-induced effects superimposed on the Minnelide transcriptional background.

### Targeted gene expression

**Fig. 5:**
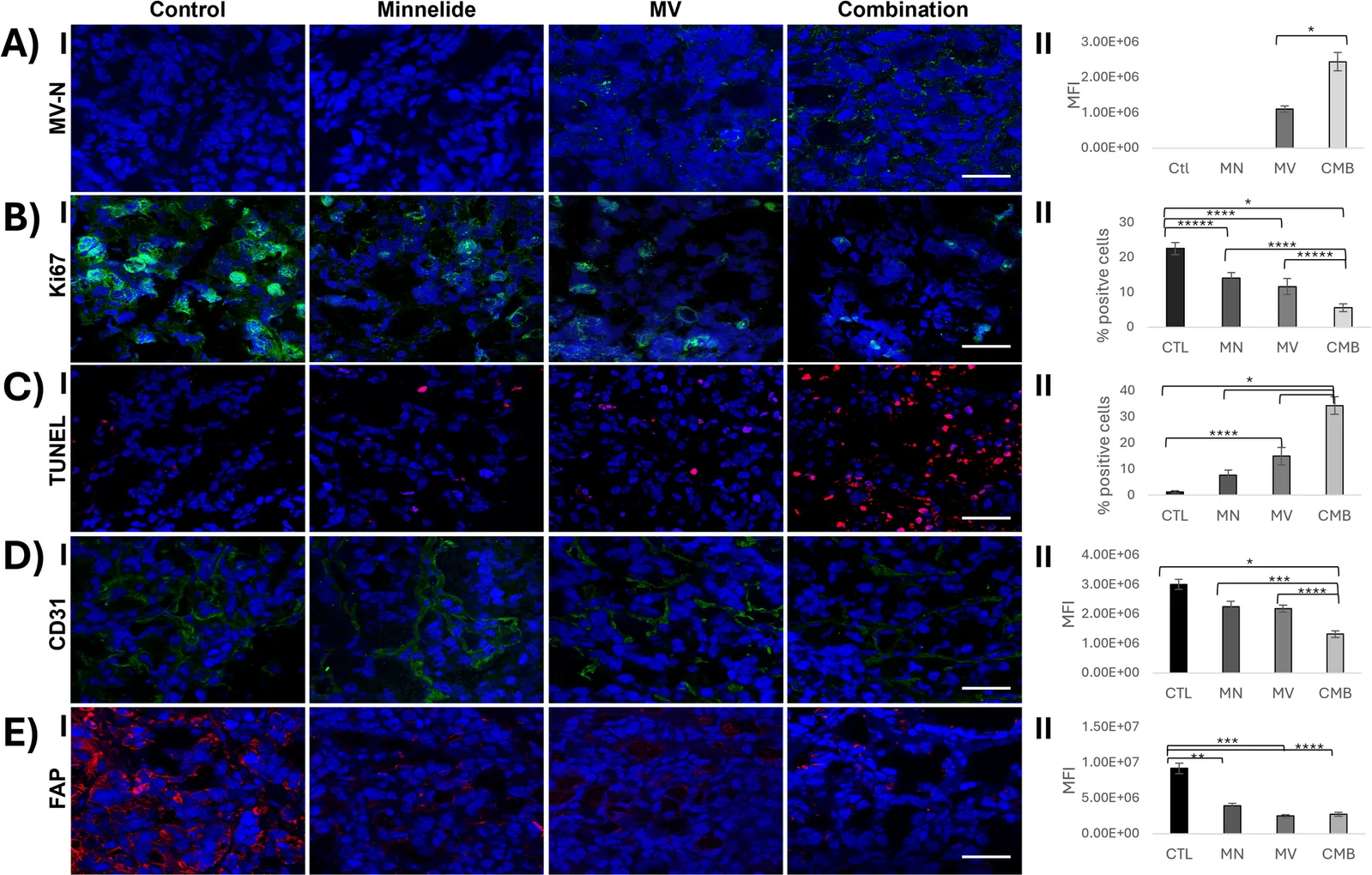
Analysis of tumor samples by Immunofluorescence (IF) studies. HT29 bearing NSG mice were treated (*n* = 8 per group) as described in the methods section. Tumors were resected at treatment day 7 and processed for tumor correlative studies. Representative immunofluorescent pictures (**I**) and quantitative analysis (**II**) of MV-N (measles virus protein, **A**, Ki67 (**B**). TUNEL (**C**), CD31 (**D**), and FAP (**E**). *p < 0.0001, **p < 0.0005, ***p < 0.005, ****p < 0.05, *****p < 0.05. Scale bar = 50 μm

Using the NanoString Human Tumor Signaling 360 panel, we further characterized the tumor signaling pathways modulated by the MV-Minnelide combination. This treatment broadly downregulated pro-survival and proliferative cascades (*PI3K*, *mTOR*, *MAPK* and *MYC*), metabolic networks (glucose, glutamine, and lipid metabolism), and interferon stimulated genes (Fig. 5. I). These effects were largely driven by MN addition. Conversely, the combination upregulated p53, apoptosis and senescence pathways more robustly than monotherapies, indicating activation of integrated stress responses leading to irreversible cell cycle arrest and apoptosis. Gene level analysis (Fig. 5. J and Supplementary Data) confirmed suppression of DNA replication and repair regulators (*WEE1*, *CDC25C*, *MCM2*, *RAD51*, *PARP* and *CHEK2*) and metabolic enzymes (*HK2*, *LDHA*, *IDH1* and *SLCA1*). Antiviral mediators (*TLR3*, *MYD88*, *STAT1/3* and *IRF1/7*) were differentially suppressed, suggesting impaired innate antiviral responses. Moreover, epithelial-mesenchymal transition (EMT) markers (*CD44*, *AKT1*, *PTEN*, *ITGB1*, *LAMC2*) hypoxia effectors (*CA9*,* EDN1* and *SERPINE1*) and extracellular matrix genes (*ITGB1* and *LAMC2*), were downregulated.

Corelative tumor studies (Fig. 5. A-E) confirmed significantly enhanced intratumoral viral distribution (MV-N), reduced proliferation (Ki67), increased apoptosis, and stromal remodeling (decreased CD31 and FAP). Together, these findings indicate that Minnelide amplifies oncolytic virus efficacy by modulating tumor intrinsic signaling, metabolic fitness, antiviral defenses, and tumor stroma.

## Discussion

Advanced colorectal cancer (CRC) remains a major therapeutic challenge due to its aggressive biology, limited durability of current treatments, and rising incidence in younger adults [41]. These challenges are particularly evident in BRAF V600E mutant tumors, where responses to targeted agents are short lived and therapeutic options after progression are limited [42, 43]. These limitations underscore the urgent need for strategies to overcome CRC treatment resistance. Oncolytic viruses represent an attractive therapeutic platform because they integrate direct oncolysis with immune activation through non-overlapping mechanisms. In this context, our dual targeted MV-CD46-muPA, designed to infect human tumor cells via CD46 and murine stromal cells through murine uPAR has shown measurable in vivo antitumor activity in CRC models [21, 44]. However, like most OVs, its efficacy as monotherapy remains limited by antiviral defenses and adaptive survival pathways that lead to tumor resistance. These observations prompted the investigation of rational combinations capable of amplifying oncolytic virus potency.

Here, we identify triptolide (TRP) and its clinical analogue Minnelide as context-dependent modulators of MV-mediated antitumor activity in human colorectal cancer models. TRP enhanced the antitumor effects of MV in multiple CRC models and experimental systems, including 2D growth assays, 3D spheroid cultures, and in vivo xenografts. In vitro, the most evident combinatorial effects were observed in the BRAF V600E-mutant HT29 and RKO models. Additional CRC models, including KRAS-mutant and non-V600 BRAF-mutant CRC cells, also demonstrated combinatorial activity, although the magnitude and kinetics of response varied across models. Notably, combinatorial effects were more consistently observed in 3D spheroid models across most CRC models tested than in the corresponding 2D assays, further supporting the biologic and context-dependent nature of MV/TRP activity across distinct CRC settings.

Although TRP increased viral RNA levels across all tested cell lines, the extent of cell death varied between models and did not uniformly parallel the degree of viral RNA expression, suggesting that additional context-dependent mechanisms may contribute to the combinatorial effects. This contrasts with prior work in vesicular stomatitis virus (VSV), where TRP-mediated enhancement of oncolytic activity was attributed primarily to inhibition of type I interferon signaling and increased viral replication [45]. Our integrated transcriptomic and proteomic analyses revealed a broader and more complex mechanism through which TRP modulates MV-mediated oncolysis, involving coordinated disruption of cellular programs that normally limit viral cytotoxicity. These alterations included enhanced inhibition of PI3K/AKT/mTOR signaling, perturbation of autophagy and DNA repair pathways, downregulation of glycolytic, mitochondrial, and anabolic metabolism, and modulation of stress, survival, and apoptosis-associated signaling networks. Collectively, these convergent molecular alterations suggest the establishment of a cellular state that is more permissive to MV-mediated antitumor activity.

The above in vitro mechanisms translated into meaningful in vivo antitumor activity that was influenced by oncogenic context. The MV‑CD46‑muPA–Minnelide combination delayed tumor progression in both models, with more pronounced and durable effects in the BRAF‑mutant HT29 setting, including in tumors with larger baseline burden. The enhanced efficacy observed in higher burden HT29 tumors may reflect increased dependence of advanced tumors on survival, metabolic, and stress‑response pathways targeted by the combination, thereby increasing permissiveness to viral oncolysis. In contrast, responses in the KRAS‑mutant HCT116 model were more modest and dose‑dependent, with combinatorial benefit less pronounced than in BRAF‑mutant tumors. These differences underscore the context‑dependent nature of the MV–Minnelide interaction and suggest that BRAF‑mutant CRC represents a more favorable biological setting for this virus-drug combination strategy. Minnelide was administered for 21 days to balance efficacy and tolerability, and while this schedule achieved meaningful tumor control without significant toxicity, regrowth following treatment cessation suggests that sustained therapeutic pressure may be required for durable responses, supporting future studies to optimize dosing duration and scheduling.

Transcriptomic profiling of HT29 tumors treated with MV plus Minnelide confirmed suppression of PI3K/AKT/mTOR, cell cycle, interferon, NF-κB, and metabolic pathways, together with activation of p53-driven stress and apoptotic programs, consistent with the in vitro findings. In addition, combination-treated tumors demonstrated reduced expression of antiviral response and extracellular matrix–associated genes. Tumor immunofluorescence analyses further supported these molecular findings, demonstrating increased intratumoral viral signal, increased apoptosis, and marked reductions in proliferation, angiogenesis, and fibroblast activation in MV plus Minnelide–treated tumors. Together, these findings suggest that Minnelide enhances MV efficacy not only through modulation of tumor-intrinsic signaling pathways, but also through treatment-associated changes in antiviral responses and stromal-associated tumor features that may facilitate intratumoral viral activity and antitumor effects. Because Minnelide broadly modulates host stress and survival pathways, its capacity to enhance viral efficacy may be relevant to other OV platforms beyond measles.

Despite strong preclinical evidence, several limitations should be noted. Our studies were conducted in human CRC xenografts, which lack functional immunity and therefore cannot fully capture the immunologic impact of the combination. Ongoing work in syngeneic, immunocompetent CRC models -including combinations with checkpoint blockade- will address this gap. We have previously shown that MV-muPA, a murine adapted MV, exhibits potent activity in murine CRC [46], placing us in a strong position to dissect immune mechanisms. Although BRAF-mutant CRC appeared to benefit most from the combination, further validation in genetically diverse models -including PDXs- will be necessary to confirm genotype specific responses. Differences in antitumor activity observed between the current study and our prior report on the activity of MV-CD46-muPA [21] may reflect differences in study design, including host age, sex distribution, and tumor burden at treatment initiation, all of which may differentially influence responsiveness to single-agent oncolytic virotherapy. Finally, while pre-existing measles immunity may influence clinical efficacy, Minnelide’s ability to transiently suppress antiviral mechanisms may enhance viral entry and replication and may further sensitize tumors to immunotherapy, when appropriately sequenced.

## Conclusion

Our findings demonstrate that Minnelide amplifies the therapeutic activity of oncolytic measles virotherapy through coordinated modulation of virus-tumor-stromal interactions. By suppressing survival, metabolic, and antiviral programs, reinforcing apoptotic signaling, and remodeling the tumor microenvironment to facilitate viral tumor entry, Minnelide creates a tumor ecosystem that is permissive to measles virus oncolysis. These multifaceted effects lead to potent and durable antitumor responses, particularly in BRAF V600E mutant CRC models. Collectively, these results provide a strong mechanistic and translational foundation for advancing Minnelide-based oncolytic virotherapies to improve outcomes in treatment refractory colorectal cancer.

## Supplementary Information


Supplementary Material 1.



Supplementary Material 2.



Supplementary Material 3.



Supplementary Material 4.



Supplementary Material 5.



Supplementary Material 6.



Supplementary Material 7. Supplementary Methods.


## Data Availability

The data have been submitted to the NCBI GEO database. Since the datasets are scheduled for release at a later date, we have generated secure review tokens in case the reviewers request access.RNA-seq: GSE312474 / Secure token for private review: kfyfogqwtpwblqfNanostring: In vivo – GSE312436 / Secure token for private review: evgfqokulzkplixNanostring: In vitro – GSE312232 / Secure token for private review: ybqfgsykfvgnpcdRPPA: GSE313341 / Secure token for private review: Qrgluwegptsppqp.

## Abbreviations

CRC: Colorectal cancer
OVs: Oncolytic viruses
BRAF: B-Raf proto-oncogene, serine/threonine kinase
KRAS: Kirsten rat sarcoma viral oncogene homolog
MV: Measles virus
uPAR: urokinase plasminogen activator surface receptor
TRP: Triptolide
MN: Minnelide
RPPA: Reverse Phase Protein Array
MSS: Microsatellite stable
DEG: Differentially expressed gene(s)

## References

[1] Siegel RL, et al. Cancer statistics, 2026. CA Cancer J Clin. 2026;76(1):e70043. 41528114 10.3322/caac.70043 PMC12798275

[2] Gutierrez ME, et al. Genomic Profiling for KRAS, NRAS, BRAF, Microsatellite Instability, and Mismatch Repair Deficiency Among Patients With Metastatic Colon Cancer. JCO Precis Oncol; 2019. p. 3. 10.1200/PO.19.00274 PMC7448804 32923867

[3] Martinelli E, et al. European expert panel consensus on the clinical management of BRAF(V600E)-mutant metastatic colorectal cancer. Cancer Treat Rev. 2023;115:102541. 36931147 10.1016/j.ctrv.2023.102541

[4] Ni Y, et al. The Role of Tumor-Stroma Interactions in Drug Resistance Within Tumor Microenvironment. Front Cell Dev Biol. 2021;9:637675. 34095111 10.3389/fcell.2021.637675 PMC8173135

[5] Khalaf K, et al. Aspects of the Tumor Microenvironment Involved in Immune Resistance and Drug Resistance. Front Immunol. 2021;12:656364. 34122412 10.3389/fimmu.2021.656364 PMC8190405

[6] Jing Y, et al. In vivo antitumor activity by dual stromal and tumor-targeted oncolytic measles viruses. Cancer Gene Ther. 2020;27(12):910–22. 32231231 10.1038/s41417-020-0171-1 PMC7529863

[7] Kaufman HL, Kohlhapp FJ, Zloza A. Oncolytic viruses: a new class of immunotherapy drugs. Nat Rev Drug Discov. 2015;14(9):642–62. 26323545 10.1038/nrd4663 PMC7097180

[8] Robinson S, Galanis E. Potential and clinical translation of oncolytic measles viruses. Expert Opin Biol Ther. 2017;17(3):353–63. 28129716 10.1080/14712598.2017.1288713 PMC5381660

[9] Msaouel P, et al. Clinical Trials with Oncolytic Measles Virus: Current Status and Future Prospects. Curr Cancer Drug Targets. 2018;18(2):177–87. 28228086 10.2174/1568009617666170222125035 PMC5630504

[10] Aref S, Bailey K, Fielding A. Measles Rescue: Rev Oncolytic Measles Virus Viruses, 2016;8(10):294. 10.3390/v8100294 PMC5086626 27782084

[11] Perez-Dominguez F, et al. Oncolytic Viruses as a Novel Therapeutic Approach for Colorectal Cancer: Mechanisms, Current Advances, and Future Directions. Cancers (Basel), 2025;17(11):1854. 10.3390/cancers17111854 PMC12153852 40507337

[12] Engeland CE, Ungerechts G. Measles Virus as an Oncolytic Immunotherapy. Cancers (Basel), 2021;13(3):544. 10.3390/cancers13030544 PMC7867054 33535479

[13] Ottolino-Perry K, et al. Intelligent design: combination therapy with oncolytic viruses. Mol Ther. 2010;18(2):251–63. 20029399 10.1038/mt.2009.283 PMC2839289

[14] Bommareddy PK, Shettigar M, Kaufman HL. Integrating oncolytic viruses in combination cancer immunotherapy. Nat Rev Immunol. 2018;18(8):498–513. 29743717 10.1038/s41577-018-0014-6

[15] Bommareddy PK, Shettigar M, Kaufman HL. Author Correction: Integrating oncolytic viruses in combination cancer immunotherapy. Nat Rev Immunol. 2018;18(8):536. 29930315 10.1038/s41577-018-0031-5

[16] Jing Y, et al. Tumor and vascular targeting of a novel oncolytic measles virus retargeted against the urokinase receptor. Cancer Res. 2009;69(4):1459–68. 19208845 10.1158/0008-5472.CAN-08-2628 PMC2739455

[17] Zhai BT, et al. Urokinase-type plasminogen activator receptor (uPAR) as a therapeutic target in cancer. J Transl Med. 2022;20(1):135. 35303878 10.1186/s12967-022-03329-3 PMC8932206

[18] Jing Y, et al. In vivo anti-metastatic effects of uPAR retargeted measles virus in syngeneic and xenograft models of mammary cancer. Breast Cancer Res Treat. 2015;149(1):99–108. 25519042 10.1007/s10549-014-3236-8 PMC4302411

[19] Jing Y, et al. Molecular Effects of Stromal-Selective Targeting by uPAR-Retargeted Oncolytic Virus in Breast Cancer. Mol Cancer Res. 2017;15(10):1410–20. 28679779 10.1158/1541-7786.MCR-17-0016 PMC5682105

[20] Jing Y, et al. In vivo safety, biodistribution and antitumor effects of uPAR retargeted oncolytic measles virus in syngeneic cancer models. Gene Ther. 2014;21(3):289–97. 24430235 10.1038/gt.2013.84 PMC3949200

[21] Jing Y, et al. In vivo antitumor activity by dual stromal and tumor-targeted oncolytic measles viruses. Cancer Gene Ther; 2020; 27(12):910–22 . 10.1038/s41417-020-0171-1 PMC7529863 32231231

[22] Lin D, Shen Y, Liang T. Oncolytic virotherapy: basic principles, recent advances and future directions. Signal Transduct Target Ther. 2023;8(1):156. 37041165 10.1038/s41392-023-01407-6 PMC10090134

[23] Larrieux A, Sanjuan R. Cellular resistance to an oncolytic virus is driven by chronic activation of innate immunity. iScience. 2023;26(1):105749. 36590165 10.1016/j.isci.2022.105749 PMC9794979

[24] Rider PJF, et al. Anti-viral immunity in the tumor microenvironment: implications for the rational design of herpes simplex virus type 1 oncolytic virotherapy. Curr Clin Microbiol Rep. 2019;6(4):193–9. 33344108 10.1007/s40588-019-00134-3 PMC7748056

[25] Ricca JM, et al. Pre-existing Immunity to Oncolytic Virus Potentiates Its Immunotherapeutic Efficacy. Mol Ther. 2018;26(4):1008–19. 29478729 10.1016/j.ymthe.2018.01.019 PMC6079372

[26] Kurokawa C, Galanis E. Interferon signaling predicts response to oncolytic virotherapy. Oncotarget. 2019;10(16):1544–5. 30899421 10.18632/oncotarget.26679 PMC6422185

[27] Bhatt DK, Chammas R, Daemen T. Resistance Mechanisms Influencing Oncolytic Virotherapy, a Systematic Analysis. Vaccines (Basel). 2021;9(10):1166. 10.3390/vaccines9101166 PMC8537623 34696274

[28] Feng K, et al. Mechanisms of cancer cell death induction by triptolide: A comprehensive overview. Heliyon. 2024;10(2):e24335. 38293343 10.1016/j.heliyon.2024.e24335 PMC10826740

[29] Oliveira A, et al. Triptolide abrogates growth of colon cancer and induces cell cycle arrest by inhibiting transcriptional activation of E2F. Lab Invest. 2015;95(6):648–59. 25893635 10.1038/labinvest.2015.46 PMC5001951

[30] Wang Z, et al. Triptolide downregulates Rac1 and the JAK/STAT3 pathway and inhibits colitis-related colon cancer progression. Exp Mol Med. 2009;41(10):717–27. 19561401 10.3858/emm.2009.41.10.078 PMC2772974

[31] Brincks EL, et al. Triptolide enhances the tumoricidal activity of TRAIL against renal cell carcinoma. FEBS J. 2015;282(24):4747–65. 26426449 10.1111/febs.13532 PMC4715782

[32] Banerjee S, et al. Impaired Synthesis of Stromal Components in Response to Minnelide Improves Vascular Function, Drug Delivery, and Survival in Pancreatic Cancer. Clin Cancer Res. 2016;22(2):415–25. 26405195 10.1158/1078-0432.CCR-15-1155 PMC4716007

[33] Chang WT, et al. Triptolide and chemotherapy cooperate in tumor cell apoptosis. A role for the p53 pathway. J Biol Chem. 2001;276(3):2221–7. 11053449 10.1074/jbc.M009713200

[34] Modi S, et al. Minnelide synergizes with conventional chemotherapy by targeting both cancer and associated stroma components in pancreatic cancer. Cancer Lett. 2022;537:215591. 35398530 10.1016/j.canlet.2022.215591

[35] Nakamura T, et al. Rescue and propagation of fully retargeted oncolytic measles viruses. Nat Biotechnol. 2005;23(2):209–14. 15685166 10.1038/nbt1060

[36] Rao X, et al. An improvement of the 2^(-delta delta CT) method for quantitative real-time polymerase chain reaction data analysis. Biostat Bioinforma Biomath. 2013;3(3):71–85. 25558171 PMC4280562

[37] Rausch M, et al. Identification of low-dose multidrug combinations for sunitinib-naive and pre-treated renal cell carcinoma. Br J Cancer. 2020;123(4):556–67. 32439932 10.1038/s41416-020-0890-y PMC7435198

[38] Varudkar N, et al. Oncolytic parainfluenza virus combines with NK cells to mediate killing of infected and non-infected lung cancer cells within 3D spheroids: role of type I and type III interferon signaling. J Immunother Cancer, 2021;9(6):e002373. 10.1136/jitc-2021-002373 PMC8237729 34172515

[39] Siwak DR, et al. Analytical Platforms 3: Processing Samples via the RPPA Pipeline to Generate Large-Scale Data for Clinical Studies. Adv Exp Med Biol. 2019;1188:113–47. 31820386 10.1007/978-981-32-9755-5_7

[40] Ahmed D, et al. Epigenetic and genetic features of 24 colon cancer cell lines. Oncogenesis. 2013;2(9):e71. 24042735 10.1038/oncsis.2013.35 PMC3816225

[41] Sung H, et al. Colorectal cancer incidence trends in younger versus older adults: an analysis of population-based cancer registry data. Lancet Oncol. 2025;26(1):51–63. 39674189 10.1016/S1470-2045(24)00600-4 PMC11695264

[42] Formica V, et al. KRAS and BRAF Mutations in Stage II and III Colon Cancer: A Systematic Review and Meta-Analysis. J Natl Cancer Inst. 2022;114(4):517–27. 34542636 10.1093/jnci/djab190 PMC9002292

[43] El Hage M, Su Z, Linnebacher M. Addressing Challenges in Targeted Therapy for Metastatic Colorectal Cancer. Cancers (Basel), 2025;17(7):1098. 10.3390/cancers17071098 PMC11988006 40227578

[44] Jessie B, Dobrovolny HM. The role of syncytia during viral infections. J Theor Biol. 2021;525:110749. 33964289 10.1016/j.jtbi.2021.110749

[45] Ben Yebdri F, et al. Triptolide-mediated inhibition of interferon signaling enhances vesicular stomatitis virus-based oncolysis. Mol Ther. 2013;21(11):2043–53. 23985699 10.1038/mt.2013.187 PMC3831042

[46] Jing Y, et al. In vivo safety, biodistribution and antitumor effects of uPAR retargeted oncolytic measles virus in syngeneic cancer models. Gene Ther. 2014;21(3):289–97. 24430235 10.1038/gt.2013.84 PMC3949200

