# Supplementary material for: Pharmacologic reprogramming of virus-tumor crosstalk enhances measles virus antitumor activity in BRAF mutant colorectal cancer models
Source: J Exp Clin Cancer Res. 2026 Jun 6;45:191. doi: 10.1186/s13046-026-03749-9 (PMC13536603; doi:10.1186/s13046-026-03749-9)
Supplement: Supplementary file 1 [file 13046_2026_3749_MOESM7_ESM.docx]

**SUPPLEMENTARY METHODS**

**RNA sequencing. Read Processing and Alignment.** The sequencing reads from xenograft samples were processed using Xenome, a tool within the Gossamer bioinformatics suite **(PMID:** [**29304755**](https://pubmed.ncbi.nlm.nih.gov/29304755/)**, PMID:** [**22689758**](https://pubmed.ncbi.nlm.nih.gov/22689758/)**)**. Xenome is designed to accurately differentiate and classify reads from both host and graft tissues, a critical step for accurately separating host and graft reads, ensuring downstream analyses focus on the correct transcriptomes. Read alignment was performed using STAR (v2.7.11b) (PMID: 23104886).

**Normalization and Differential Gene Expression Analysis.** Count normalization and differential gene expression (DEG) analysis were carried out using the DESeq2 pipeline (**PMID: 25516281**), enabling the identification of genes with altered expression between the control and drug-treated groups: (1) Control vs MV, (2) Control vs Minnelide, and (3) Control vs Combination (MV + Minnelide). To correct for variations in sequencing depth and library size across samples, the data were normalized using variance stabilizing transformation (VST), implemented in the DESeq2 package within the R environment **(**[**https://www.R-project.org/**](https://www.R-project.org/)**).** .

To control for false discoveries, p-values were adjusted using the Benjamini-Hochberg method, which controls the false discovery rate (FDR). A threshold of adjusted p-value < 0.05 was applied to ensure statistical significance, along with a log2 fold change ≥ 1 to identify both up and down regulated genes with biologically relevant changes in expression.

**Gene Set Enrichment Analysis (GSEA).** To assess pathway-level changes in gene expression, we performed Gene Set Enrichment Analysis (GSEA) using the clusterProfiler R package (PMID: 22455463). Hallmark gene sets (category H), canonical pathways (C2, subcategory CP), and Gene Ontology terms (C5) were retrieved from the Molecular Signatures Database (MSigDB) via the msigdbr R package (PMID: **26771021**). Gene sets were formatted into TERM2GENE data frames to map genes to corresponding pathways.

Genes were ranked in decreasing order of log2 fold change (combination treatment vs. control) and aggregated by gene symbol before performing GSEA. GSEA was then performed using the ranked gene list with the GSEA() function from clusterProfiler, using the Benjamini-Hochberg method to adjust p-values for multiple testing. Enrichment was tested against the **Hallmark gene sets** (category H) from the **Molecular Signatures Database (MSigDB v7.5.1)** (Liberzon et al., 2015). Normalized Enrichment Scores (NES) of enriched pathways were calculated to adjust for gene set size, and statistical significance was assessed using **Benjamini–Hochberg (BH)** adjusted p-values. Pathways with significantly positive NES values were considered **upregulated,** whereas those with negative NES values were considered **downregulated** relative to control.

Visualization of enriched pathways was performed using dot plots, highlighting normalized enrichment scores and adjusted p-values. Core enrichment genes contributing to each pathway are reported in Supplementary Table 1.

**Ingenuity Pathway Analysis (IPA)s**. We employed QIAGEN’s Ingenuity® Pathway Analysis (IPA®) software (QIAGEN Redwood City, www.qiagen.com/ingenuity) to identify the most significant canonical pathways, diseases, and functions, and gene networks related to DESeq2 outcomes, and to categorize differentially expressed genes according to specific diseases and functions. Genes were categorized into biological functions based on the Ingenuity knowledge base, and z-scores were calculated to predict whether each function was activated or inhibited. Enrichment of disease and biological function categories was assessed using a standard IPA Core Analysis with default parameters; significance was determined by right-tailed Fisher's exact test and expressed as –log(p-value), with –log(p-value) ≥ 1.3 (p ≤ 0.05) considered significant.

**Targeted gene expression analysis (Nanostring).** RNA was extracted as described above from HT-29 cell lines treated with MV, Triptolide or the combination, as well as HT-29 treated and control tumors, as described above. Targeted gene expression analysis was performed from samples using nCounter XT Hs Tumor Signaling 360 (nanoString, 115000409) panels run on a nCounter Pro analysis system following manufacturer’s instructions, with RNA inputs of 100 ng at >DV200 (126 – 720 ng total RNA) and 16hr probe hybridization times. Cartridges were scanned in high sensitivity mode. Data was obtained using NanoString's (NanoString Technologies, USA) nCounter Raw Code Count Collector tool and imported into their nSolver 4.0 software. Raw data was processed and analyzed using nSolver and the nSolver Advanced Analysis software v2.0.134 under standard settings. Differential expression analysis was conducted after normalization against the manufacturer’s default housekeeping genes. Advanced analysis settings were set as recommended by the manufacturer, with p-value adjustments applied through the Benjamini-Hochberg procedure. Heatmaps were generated from the nSolver platform filtering for statistically significant genes (p≤0.05) and gene sets of interest in the Tumor Signaling panel.
